# Neurodynamical model for visual action recognition

**DOI:** 10.1186/1471-2202-15-S1-P164

**Published:** 2014-07-21

**Authors:** Martin A Giese, Leonid Fedorov

**Affiliations:** 1 Section Computational Sensomotorics, HIH / CIN, University Clinic Tübingen, Germany

## 

The recognition of body motion requires the temporal integration information over time. This integration likely is achieved by dynamic neural networks, which are composed from neurons that are selective for form and optic flow patterns [1]. Physiological evidence also indicates that many action-selective visual neurons are view-dependent, so that such representations likely represent not only the time structure of actions, but also the stimulus view. The dynamic and self-organization properties of such representations have rarely been studied.

We propose a neurodynamical model for the visual encoding of actions that is based on a two-dimensional neural field with the defining equations

$$\begin{array}{c} \tau_{u}\dot{u}\left(\varphi ,\theta ,t\right)=-u\left(\varphi ,\theta ,t\right)+w\left(\varphi ,\theta \right)*{\left[u\left(\varphi ,\theta ,t\right)\right]}_{+}+s\left(\varphi ,\theta ,t\right)-\alpha a\left(\varphi ,\theta ,t\right)+\xi \left(\varphi ,\theta ,t\right)\\ \tau_{a}ȧ\left(\varphi ,\theta ,t\right)=-a\left(\varphi ,\theta ,t\right)+{\left[u\left(\varphi ,\theta ,t\right)\right]}_{+} \end{array}$$

*u *specifies the membrane potential and *a *the adaptation state. The recurrent interaction kernel *w *is symmetric with respect to the origin in ø-direction and asymmetric in ξ-direction, with an additional strong inhibitory component. The spatial convolution * is periodic. The stimulus signal *s *models an (idealized) activity distribution of shape- (or optic flow-) selective neurons that are maximally responding to stimulus frame θ and view angle ø of an ongoing action stimulus. The noise variable ξ is defined by a Gaussian process whose kernel was fitted in order to reproduce coarsely the correlation statistics, dependent on the tuning similarity of the neurons. Time scales and adaptation strength are specified by the positive constants *τ_u_*, *τ_v_*and α. The first equation defines a dynamic neural field that stabilizes a stimulus-locked travelling peak solution in ξ-direction, and a winner-takes-all competition in ø-direction. The second equation specifies a simple adaptation process.

## Results

The model accounts simultaneously for a variety of dynamic properties of visual action-selective neurons and visual body motion perception: a) Temporal sequence selectivity, as observed in the STS and area F5 (e.g. [2]). b) Bistable perception of biological motion with spontaneous perceptual switching between multiple perceived heading directions from walker silhouettes [3]. The model allows to investigate the roles of adaptation and noise in such perceptual switching. c) Prediction of a bifurcation between a single stable and two competing travelling pulse solutions dependent on the view angle of the stimulus. d) The comparison of adaptation for static and dynamic adaptator stimuli provides possible explanation for the observed lack of adaptation in action-selective neurons (e.g. [4]). The adaptation characteristics reproduces the behavior of neurons in area IT ('input fatigue model') [5].

## Conclusions

The new model provides a basis for the quantitative study of dynamic and self-organization phenomena in action perception. Simplicity of the model makes it accessible for mathematical analysis, e.g. of bifurcations.

## References

[B1] Giese MA Poggio T Neural mechanisms for the recognition of biological movements Nat Rev Nsc 2003 15 3 179 192 12612631 10.1038/nrn1057

[B2] Barraclough NE Keith RH Xiao D Oram MW Perrett DI Visual adaptation to goal-directed hand actions J Cogn Neurosci 2009 15 1806 1820 18855549 10.1162/jocn.2008.21145

[B3] Vanrie J Dekeyser M Verfaillie K Perception Bistability and biasing effects in the perception of ambiguous point-light walkers Perception 2004 15 5 547 560 10.1068/p5004 15250660

[B4] Caggiano V Mirror neurons in monkey area F5 do not adapt to the observation of repeated actions Nat Commun 2013 15 1433 23385578 10.1038/ncomms2419

[B5] De Baene W Vogels R Effects of adaptation on the stimulus selectivity of macaque inferior temporal spiking activity and local field potentials Cereb Cortex 2010 15 9 2145 2165 10.1093/cercor/bhp277 20038542

